# Discovery of Lipid Metabolism-Related Genes for Predicting Tumor Immune Microenvironment Status and Prognosis in Prostate Cancer

**DOI:** 10.1155/2022/8227806

**Published:** 2022-09-05

**Authors:** Ying Zhang, Xiangyu Kong, Shiyong Xin, Liangkuan Bi, Xianchao Sun

**Affiliations:** ^1^Department of Urology, The Second Affiliated Hospital of Anhui Medical University, Hefei 230032, China; ^2^Center of Gallbladder Disease, Shanghai East Hospital, School of Medicine, Tongji University, Shanghai 200120, China; ^3^Department of Urology, Shanghai East Hospital, School of Medicine, Tongji University, Shanghai 200120, China

## Abstract

**Background:**

Reprogramming of lipid metabolism is closely associated with tumor development, serving as a common and critical metabolic feature that emerges during tumor evolution. Meanwhile, immune cells in the tumor microenvironment also undergo aberrant lipid metabolism, and altered lipid metabolism also has an impact on the function and status of immune cells, further promoting malignant biological behavior. Consequently, we focused on lipid metabolism-related genes for constructing a novel prognostic marker and evaluating immune status in prostate cancer.

**Methods:**

Information about prostate cancer patients was obtained from TCGA and GEO databases. The NMF algorithm was conducted to identify the molecular subtypes. The least absolute shrinkage and selection operator (Lasso) regression analysis was applied to establish a prognostic risk signature. CIBERSORT algorithm was used to calculate immune cell infiltration levels in prostate cancer. External clinical validation data were used to validate the results.

**Results:**

Prostate cancer samples were divided into two subtypes according to the NMF algorithm. A six-gene risk signature (PTGS2, SGPP2, ALB, PLA2G2A, SRD5A2, and SLC2A4) was independent of prognosis and showed good stability. There were significant differences between risk groups of patients with respect to the infiltration of immune cells and clinical variables. Response to immunotherapy also differed between different risk groups. Furthermore, the mRNA expression levels of the signature genes were verified in tissue samples by qRT-PCR.

**Conclusion:**

We constructed a six-gene signature with lipid metabolism in prostate cancer to effectively predict prognosis and reflect immune microenvironment status.

## 1. Introduction

Prostate cancer (PCa) has become the second most common malignant tumor in men worldwide in terms of incidence and mortality, which seriously endangers men's health [1]. PCa is the most diagnosed cancer in men in more than half of the countries in the world, especially in developed countries and regions [2]. A large number of epidemiological studies have been conducted to confirm that age, race, and family genetic history are recognized risk factors [3]. In particular, along with the change in people's lifestyle and diet habits, obesity and the consequent disorder of blood lipid levels have been noticed. High-calorie food and saturated animal fat intake are associated with increased PCa incidence [4, 5].

Lipids, as important active molecules in cellular life activities, play an important role in adaptive changes in cancer cell metabolism [6]. Altered lipid metabolism is one of the most significant metabolic changes in tumorigenesis. Enhanced lipid synthesis or uptake contributes to the rapid growth of cancer cells and tumor formation [7, 8]. Lipids are a highly complex class of biomolecules that not only form the structural basis of biological membranes but also act as signaling molecules and energy sources. Although most somatic cells derive their lipids from food sources or hepatic synthesis, various cancers reactivate fatty acids (FA) synthesis from scratch, making them more independent of externally supplied lipids [9]. Consequently, blocking lipid supply might have a significant impact on bioenergetics, membrane biosynthesis, and intracellular signaling processes in cancer cells. In addition, altered lipid effectiveness would also affect cancer cell migration, induction of angiogenesis, metabolic symbiosis, evasion of immune surveillance, and cancer drug resistance [10, 11]. However, targeting this aspect of cancer cell metabolism remains challenging given the complexity of cellular lipid species and the dynamic nature of their synthesis, remodeling, and catabolism.

Currently, immune cells in the tumor microenvironment (TME) also undergo lipid reprogramming, which has a significant impact on T cell function [12, 13]. Through continuous exploration and in-depth analysis, there are many new advances in the understanding of the complexity of lipid metabolism in different tumor immune cells, and the molecular mechanisms of lipid metabolism on cell function [14]. Targeting genes and enzymes related to tumor and immune lipid metabolism may have different effects on cancer prevention and treatment [15]. Therefore, abnormal lipid metabolism and tumor immunity are gaining widespread attention and enthusiasm from researchers.

In this study, the expression of lipid metabolism-related genes in PCa was examined in order to recognize hub genes that are predictive of patient outcome and immune microenvironment status. We constructed and validated a six-gene signature that accurately predicts PCa patient prognosis, along with immune infiltration cell patterns. Clinical application of this prognostic signature may be possible and reflects the immune status of PCa patients.

## 2. Materials and Methods

### 2.1. Data Collection

Human lipid metabolism pathways were downloaded from the Molecular Signature Database (MSigDB) [16], and 776 genes (Supplementary Table S1) were obtained from six lipid metabolism pathways (Supplementary Table S2). PCa samples and corresponding clinicopathological information were obtained from TCGA database and GEO database (GSE116918). The sample information in TCGA dataset was shown in Supplementary Table S3.

### 2.2. Molecular Subtype Identification

A total of 776 genes from TCGA dataset were extracted and genes with significant differential expression were selected. PCa samples were clustered using nonnegative matrix factorization (NMF) clustering algorithm [17]. We set the number of clusters *k* from 2 to 10, and determined the average contour width of the common member matrix using the *R* package “NMF.”

### 2.3. Gene Set Variation Analysis (GSVA)

The GSVA enrichment score of the signaling pathway in each PCa sample was calculated using the “GSVA” *R* package. The correlation between the different risk subgroups and clinical variables was analyzed by the chi-square test. Kaplan–Meier survival analysis was applied to analyze the difference in progression-free survival (PFS) between the two subgroups.

### 2.4. A Comprehensive Analysis of Immune Characteristics

PCa samples were examined for their immune profiles by importing their expression data into CIBERSORT and iterating 1000 times to estimate the relative proportions of immune cells. Our results were displayed as a landscape map showing the proportion of immune cells and clinicopathological factors. An immunophenoscore (IPS) was used to represent tumor immunogenicity on a scale from 0 to 10. Higher IPS scores represent increased immunogenicity. The IPS of TCGA patients was obtained from the Cancer Immunome Atlas (TCIA) (https://tcia.at/home).

### 2.5. Clinical Patients and Prostate Specimens

Sixty paired normal and tumor tissues were collected from PCa patients who underwent surgery at the Second Affiliated Hospital of Anhui Medical University (Hefei, China). They had diagnostic criteria according to the WHO classification and received no preoperative treatment. Informed consent was obtained from each patient before inclusion in the study, and ethical approval was obtained from the Ethics Committee of the Second Affiliated Hospital of Anhui Medical University.

### 2.6. RNA Extraction and qRT-PCR

TRIzol (Invitrogen, USA) was used to extract the total RNA. qRT-PCR was conducted based on the manufacturer's instruction. GAPDH was an internal control. Fold-changes were calculated by the 2^−ΔΔCt^ method. Primer information is shown in Supplementary Table S4.

### 2.7. Statistical Analysis

Bioinformatic analyses were conducted using *R* version 4.1.1. For comparing continuous data, Student's *t* or Wilcoxon test were used. The chi-square test and Fisher test were used for comparing clinical and pathological parameters. Spearman correlation analysis was used to analyze the correction between the risk signature and immune cells. All statistical *p*-values were two-sided and *p* < 0.05 was considered statistically significant.

## 3. Results

### 3.1. Different Subtypes Were Classified Based on Lipid Metabolism-Related Genes

**Figure 1:**
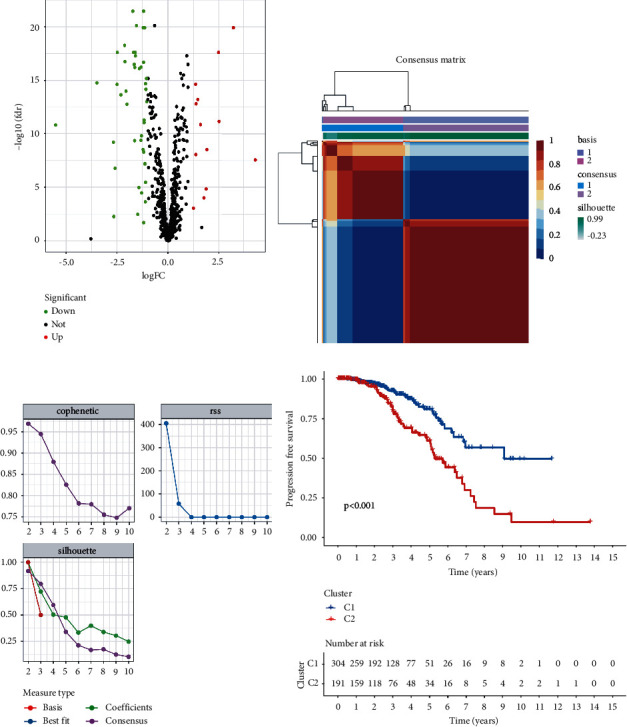
Classification was based on different subtypes. (a) Volcano map displaying the differentially expressed lipid metabolism-related genes in TCGA-PRAD. Red: up-regulation, blue: down-regulation. (b) NMF clustering consensus map. (c) NMF distributions when rank = 2–10. (d) Progression-free survival analysis of two subtypes in TCGA-PRAD.

Six lipid metabolism-related gene sets were selected from MSigDB. The gene expression of PCa was investigated using RNA-seq data from TCGA prostate cancer cohort (TCGA-PRAD). To identify genes with differential expression, the “limma” *R* package was used. The differential expression of 56 lipid metabolism-related genes were found on PCa (*p* < 0.05, Figure 1(a), Supplementary Table S5). After that, PCa samples were clustered by the NMF method. Cophenetic, dispersion, and silhouette all indicate that *k* = 2 is an optimal number of clusters (Figures 1(b) and 1(c)). PFS prognostic relationships between Cluster 1 (C1) and Cluster 2 (C2) show that subgroup C1 has a better prognosis than subgroup C2 (Figure 1(d), log-rank *p* < 0.001).

### 3.2. Establishment of the Prognostic Risk Model

**Figure 2:**
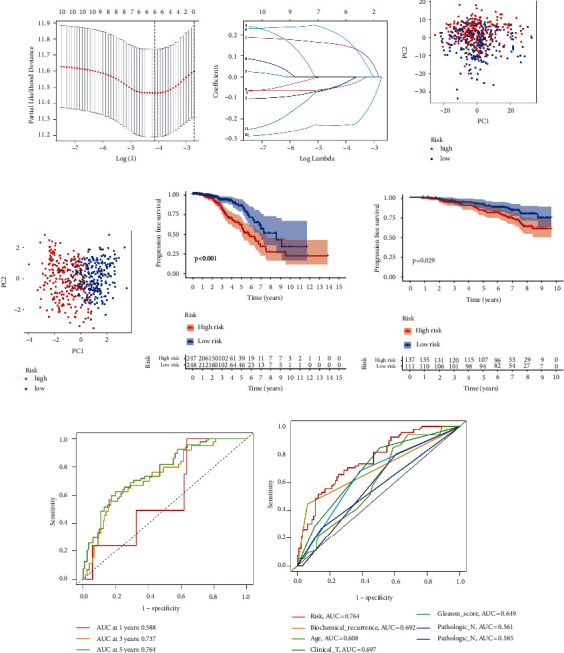
Establishment of the prognostic risk model. (a) LASSO coefficient profile plot. (b) The values of lambda in the model. (c) PCA plot in the two risk groups with lipid metabolism-related genes. (d) PCA plot in the two risk groups with risk signature genes. (e) Survival curves of the groups in TCGA-PRAD cohort. (f) Survival curves of the groups in the GSE116918 cohort. (g) ROC curve of the model in TCGA-PRAD cohort. (h) The accuracy of the risk model was tested with other clinical variables.

**Figure 3:**
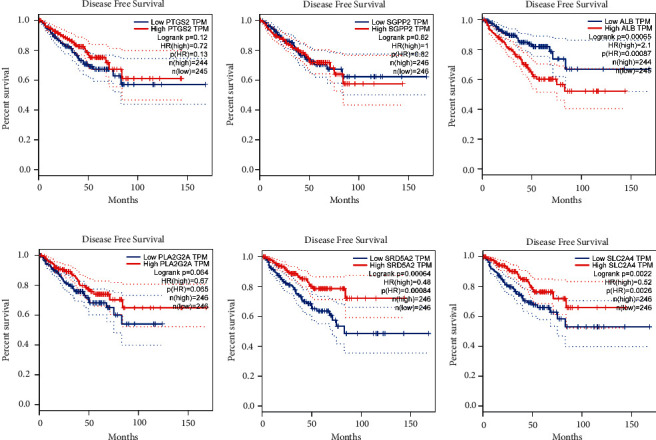
GEPIA survival analysis of PTGS2, SGPP2, ALB, PLA2G2A, SRD5A2, and SLC2A4.

To screen for significant genes associated with prognosis in TCGA-PRAD cohort, we performed a Cox proportional hazard analysis. On the basis of a *p*-value of less than 0.05, 11 genes showed significant prognostic differences (Supplementary Table S6). In order to develop a highly accurate prognostic model and to narrow the list of genes, Lasso regression analysis was used to identify hub genes (Figures 2(a) and 2(b)). Combining the analysis, six target genes were selected. The six-gene signature formula was as follows: Risk score = expression level of PTGS2 × (−0.033) + expression level of SGPP2 × (0.188) + expression level of ALB × (0.149) + expression level of PLA2G2A × (−0.045) + expression level of SRD5A2 × (−0.229) + expression level of SLC2A4 × (−0.035). PCA plot analysis demonstrated that samples in two risk groups were distributed in two directions with the six genes in our risk model compared with lipid metabolism-related gens (Figures 2(c) and 2(d)). The K-M curves for the two subgroups of the risk score were shown in Figure 2(e), and there was a significant difference between them (*p* < 0.001). We then used the same coefficients in GSE116918 as an independent validation cohort and significantly different results were observed (Figure 2(f), *p*=0.029). The area under the curve (AUC) values for 1, 3, and 5 years, respectively, were 0.588, 0.737, and 0.764 (Figure 2(g)). Furthermore, we compared the 5-year ROC curve with some clinicopathological variables. We found that the risk model exhibited satisfactory prognostic accuracy with regards to age, biochemical recurrence, clinical T stage, Gleason score, pathological N stage, and pathological T stage (Figure 2(h)). Gene Expression Profiling Interactive Analysis (GEPIA) database was applied to analyze the associations between the six signature genes and PFS in PCa [18]. Low expressions level of PLA2G2A, SRD5A2, and SLC2A4 as well as high expression level of ALB were closely correlated with poorer survival outcomes of PCa patients (Figure 3).

### 3.3. Independent Prognostic Analysis and Construction of the Nomogram

**Figure 4:**
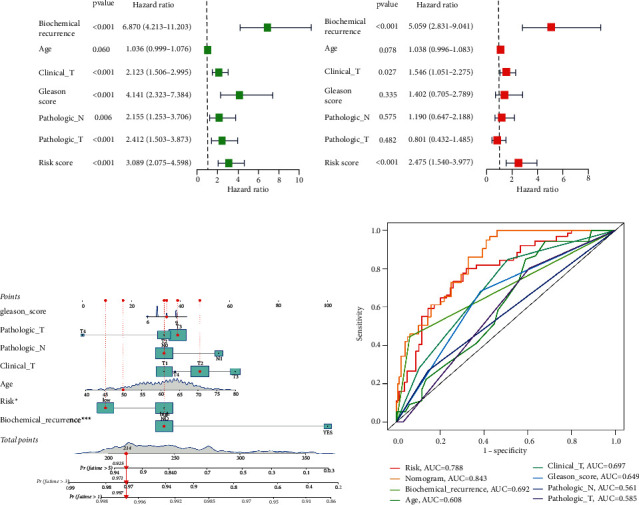
Independent prognostic analysis and construction of the nomogram. Univariate (a) and multivariate (b) cox analysis indicate that this risk signature was an independent risk factor for predicting prognosis. (c) A nomogram to predict survival. (d) AUC for the nomogram, risk, and clinical variables.

Univariate Cox regression analysis indicated that biochemical recurrence, clinical T stage, Gleason score, pathological N stage, pathological T stage. and risk were closely related to PFS (Figure 4(a)). According to multivariate analysis, only biochemical recurrence (HR = 5.059, 95% CI = [2.831–9.041], *p* < 0.001), clinical T stage (HR = 1.546, 95% CI = [1.051–2.275], *p*=0.027), and risk score (HR = 2.475, 95% CI = [1.540–3.977], *p* < 0.001) were significantly related to PFS (Figure 4(b)). These results demonstrated that this six-gene signature was an independent factor predicting prognosis. The clinicopathological features and risk were combined to construct a nomogram to assess the clinical utility of the prognostic model (Figure 4(c)). Moreover, the nomogram displayed the highest accuracy in predicting survival (AUC = 0.843) compared with other independent factors (Figure 4(d)).

### 3.4. Association between the Risk Model with Clinical Characteristics

**Figure 5:**
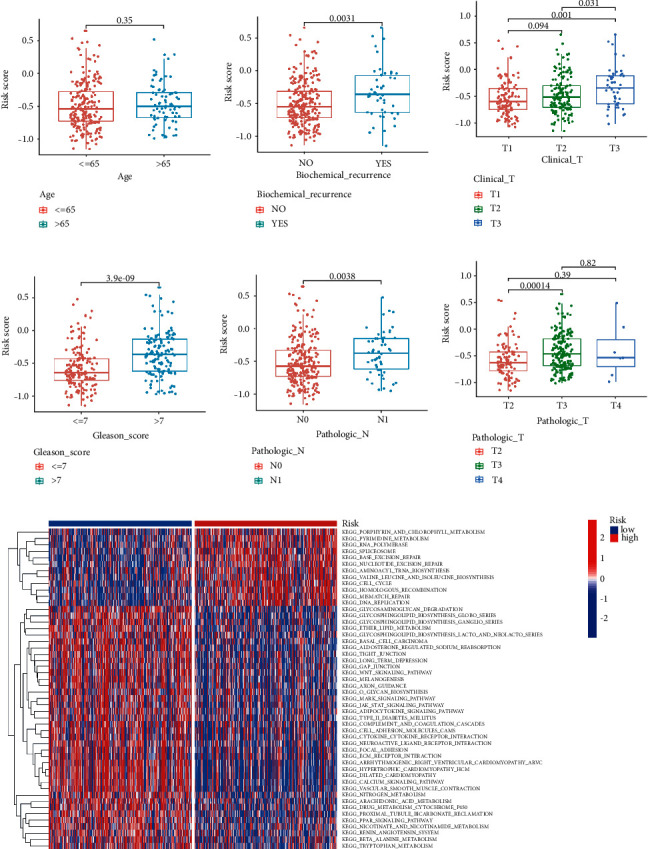
Association between the risk model with different clinical characteristics. Correlation analysis of risk with age (a), biochemical recurrence (b), clinical T stage (c), Gleason score (d), pathological N stage (e), and pathological T stage (f). (g) GSVA enrichment analysis of biological activities between the two risk groups.

Correlation analysis of the risk score and clinical variables such as biochemical recurrence, clinical T stage, Gleason score, pathological N stage, and pathological T stage indicated a statistically significant association (Figures 5(a)–5(f)). Based on the risk score, PCa patients can also be distinguished by age, clinical T stage, pathologic T stage, and N stage (Supplementary Figure S1). In addition, GSVA further confirmed that a high-risk subgroup was significantly enriched in porphyrin and chlorophyll metabolism and pyrimidine metabolism (Figure 5(g)).

### 3.5. Correlation between the Risk Model and Immunity

**Figure 6:**
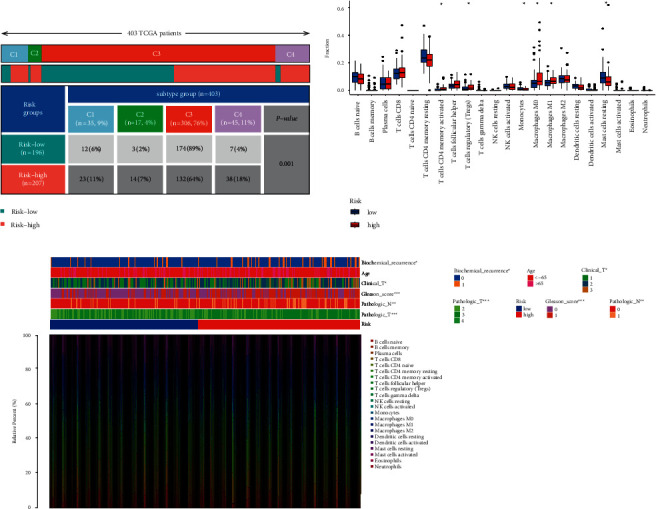
Correlation between the risk model and immunity. (a) Different immune subtypes are distributed in the two risk groups. (b) Two risk subgroups with different proportions of immune cells. (c) Clinical features of two risk subgroups with the immune landscape. ^*∗*^*p* < 0.05, ^*∗∗*^*p* < 0.01, ^*∗∗∗*^*p* < 0.001.

A 33 diverse cancer immune subtype classification has described the immune landscape of PCa according to the immune expression characteristics of four representative signatures: C1 (wound healing), C2 (IFN-*γ* dominant), C3 (inflammatory), and C4 (lymphocyte depleted) [19]. We found that a higher proportion of C1, C2, and C4 was distributed in the high-risk subgroup, while a higher proportion of C3 in the low-risk subgroup (*p*=0.001, chi-square test; Figure 6(a)). CIBERSORT was applied to evaluate the relative proportions of 22 kinds of immune cells in the TME to examine the indicative roles of this risk model [20]. A significant correlation was found between high-risk subgroups and CD4 memory-activated T cells, regulatory T cells (Tregs), M0 macrophages, and M1 macrophages, while the low-risk subgroup was significantly associated with monocytes and mast resting cells (Figure 6(b)).  Figure 6(c) illustrated the relationship between clinical and immunological characteristics of different subgroups at risk.

**Figure 7:**
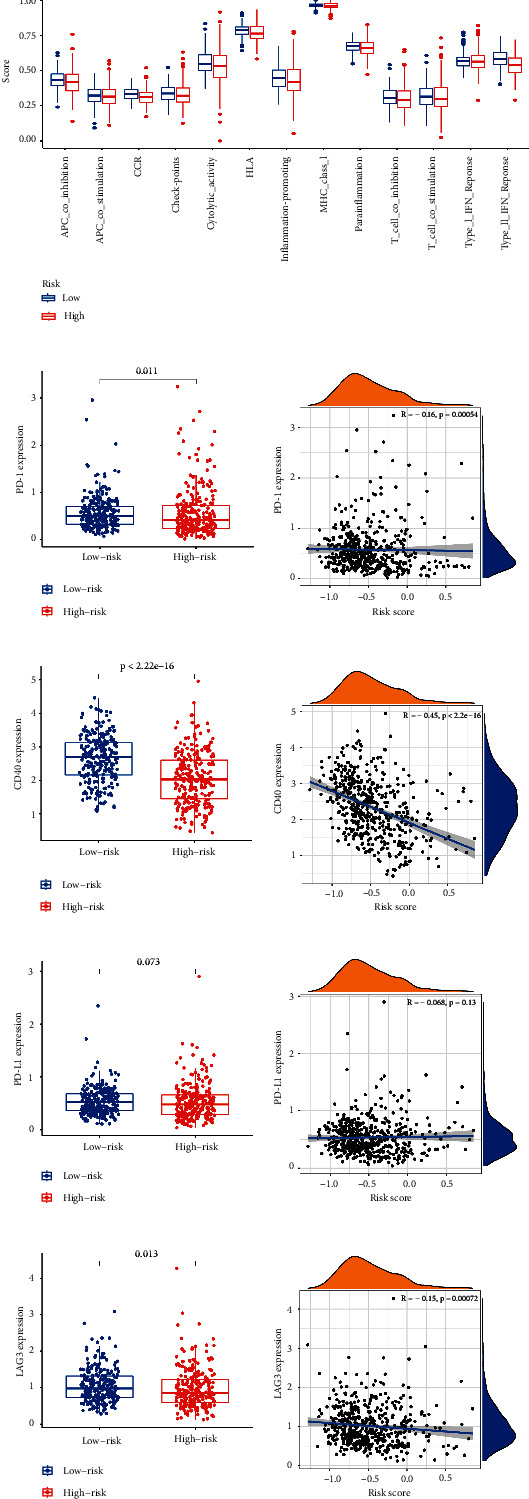
Correlation between the risk model and ICIs. (a) Immune-related pathways were quantified in different risk subgroups. Correlation between risk and PD-1 (b, c), CD40 (d, e), PD-L1 (f, g), and LAG3 (h, i). ^*∗*^*p* < 0.05, ^*∗∗*^*p* < 0.01, ^*∗∗∗*^*p* < 0.001.

As well, we investigated the potential of the risk model for predicting the response to immune checkpoint inhibitors (ICIs). The expression of PD-1, PD-L1, LAG3, and CD40 was markedly higher in the low-risk subgroup, indicating a negative correlation with risk (Figures 7(b)–7(h)). A quantification of enrichment scores of immune-related pathways was also performed. Antigen presentation functions, such as APC co-inhibition, CCR, and HLA, tended to favor the low-risk group (Figure 7(a)).

### 3.6. Role of the Risk Signature in Immunotherapeutic Responses

**Figure 8:**
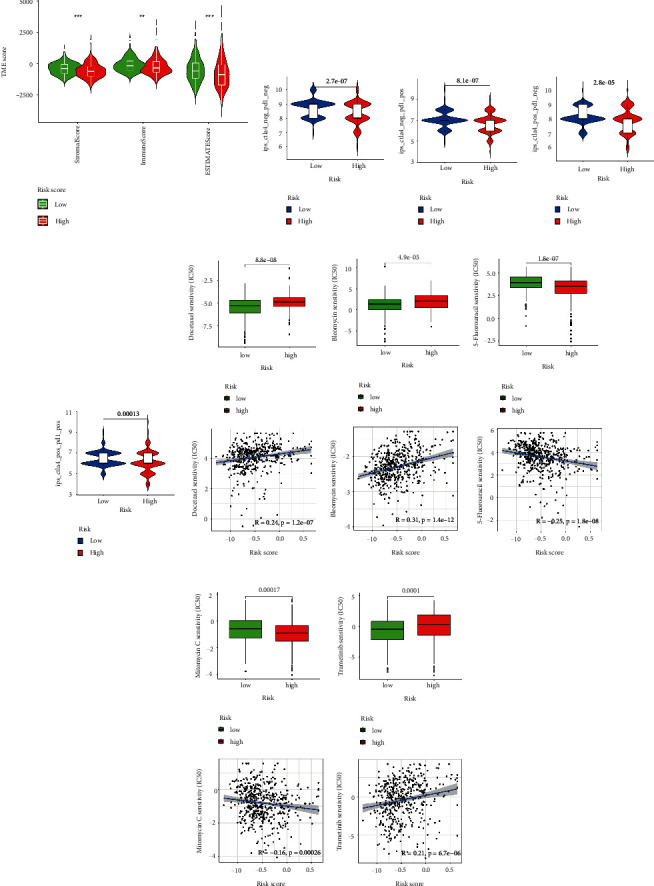
Role of the risk signature in immunotherapeutic responses. (a) ESTIMATE algorithm was used to investigate the correlation between the two groups in immune scores and stromal scores. (b)–(e) The correlation between immunophenoscore and different risk groups. Low-risk subgroup was positively correlated with a lower IC50 of Docetaxel (f), Bleomycin (g), and Trametinb (j), while a higher IC50 of 5-Fluorouracil (h) and Mitomycin C (i). ^*∗∗*^*p* < 0.01, ^*∗∗∗*^*p* < 0.001, ns *p* > 0.05.

Next, the ESTIMATE algorithm was used to investigate the correlation between the two groups in immune scores and stromal scores [21]. We found that the low-risk subgroup showed higher immune scores, stromal scores, and estimate scores than the high-risk subgroup (Figure 8(a)). These results further demonstrate that the risk model can affect the immune activity of the TME in PCa. For investigating the capacity of risk predicting response to immunotherapeutic, immunophenogram analysis was undertaken to investigate the association between immunophenoscore (IPS) and different risk subgroups [22]. Findings showed that the low-risk subgroup exhibited higher IPS compared with the high-risk subgroup, which implied that low-risk score patients exhibited a higher positive response to immunotherapy (Figures 8(b)–8(e)). Chemotherapy is an effective strategy for cancer treatment. We further analyzed the correlation between risk score and chemotherapeutic efficacy. We found that the low-risk subgroup was positively associated with a lower IC50 of Docetaxel, Bleomycin, and Trametinb, while a higher IC50 of 5-Fluorouracil and Mitomycin C, indicating a different distribution of targeted IC50 agents in low- and high-risk subgroups (Figures 8(f)–8(j)).

### 3.7. Clinical Validation of this Risk Model

**Figure 9:**
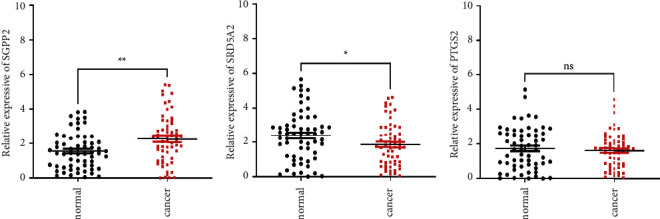
Clinical validation of this risk model. qRT-PCR analysis of SGPP2, SRD5A2, and PTGS2 mRNA levels in tissue samples (a)–(c). ^*∗*^*p* < 0.05, ^*∗∗*^*p* < 0.01, ns *p* > 0.05.

In addition to the above results, 60 cases of tissue specimens of PCa were analyzed. We verified the mRNA expression of three signature genes in cancer and normal tissues by qRT-PCR. The findings also showed that the mRNA expression of SGPP2 was higher in tumor tissues, whereas the mRNA expression of SRD5A2 was higher in normal tissues (Figure 9). These results confirmed the significant role of these genes in PCa. The workflow of the present study was shown in Supplementary Figure S2.

## 4. Discussion

PCa is becoming a growing problem among men worldwide. Its treatment is mainly divided into endocrine therapy and surgery [23]. For patients with advanced PCa, androgen resistance usually occurs, resulting in castration-resistant prostate cancer (CRPC), which severely affects the life expectancy and quality of patients [24]. Additionally, its tendency to invade surrounding tissues and cause local adhesion greatly increases the difficulty of surgery [25]. Hence, studies are continuously conducted to address the progression and aggressiveness of PCa in order to explain the pathogenesis and explore new therapeutic targets.

In terms of metabolic studies, PCa has remarkable heterogeneity. On the one hand, its metabolic pattern is different from other tumors, and on the other hand, its own metabolic form has significant phenotypic changes as the disease progresses [26, 27]. It has been clearly suggested that in prostate malignancy cells, *β*-oxidation of FA becomes one of the most important forms of energy supply [28]. Lipid accumulation and disorders of lipid metabolism in PCa cells increase the pathological process, CRPC, and aggressiveness [29]. Further understanding of the energy metabolism of PCa will enable us to design and find better drugs to prevent the development of CRPC.

Alterations in lipid metabolism affect a variety of cellular functions, which in turn affect downstream signaling pathways, associated with cell proliferation, adhesion, and motility. These alterations in the tumor can be closely related to enhanced oncogene signaling pathways and alterations in related metabolic enzymes. Moreover, the interaction between parenchyma and mesenchyme in the malignant development of tumor continuously remodels TME, and a unique tumor-associated lipid microenvironment gradually forms around it, which can have complex interactions with tumor cells through bioactive molecules such as hormones and adipokines [30, 31].

Lipid metabolism reprogramming can significantly affect immune cell fate and function. Under normal conditions, FA synthesis and uptake are key features of effector T cells. To survive in a hostile environment, immune cells undergo metabolic reprogramming, using FA as a secondary resupply station for energy [32]. FA catabolism also improves CD8^+^ T cell function through alternative pathways [33]. The normal function of immune cells is dependent on cholesterol and membrane cholesterol levels control the number of T cell receptor nanoclusters and affect their immune recognition function. Hossain et al. found that increased FA uptake and oxidation in tumor-infiltrated MDSC were accompanied by increased oxygen consumption rates and mitochondrial mass [34]. There is a potential impact of altered lipid metabolism in tumor immunity on natural killer T cell (NKT) nondependent and dependent immune function [35]. Both the M1 and M2 phenotypes of macrophages are dependent on specific lipid mediators [36].

The present study identified two subtypes of PCa based on genes that were associated with lipid metabolism using the NMF algorithm. Next, Lasso regression analysis was performed to construct a six-gene prognostic risk model. According to our study, this model performed well in predicting survival in PCa patients and correlated with both clinical features and immune microenvironment. The risk model was established with PTGS2, SGPP2, ALB, PLA2G2A, SRD5A2, and SLC2A4. Based on the corresponding coefficients, a risk score was calculated. Samples were grouped according to their risk levels. Discrepancies between the survival analyses for different risk subgroups were significant. Additionally, we found that the risk score was an independent factor for survival. CIBERSORT confirmed that patients in the high-risk subgroup had higher proportions of CD4 memory-activated T cells, regulatory T cells, M0 macrophages, and M1 macrophages, while monocytes and mast resting cells were upregulated in the low-risk group, suggesting different patterns of infiltration among the subgroups. We also demonstrated that the low-risk subgroup was correlated with immune checkpoints such as PD-1, PD-L1, CD40, and LAG3, indicating that patients with different risks respond differently to immunotherapy and low-risk patients may have a better response to immunotherapy. Next, we further validated the expression of the risk signature genes in PCa tissue specimens. The qRT-PCR results suggested that the expression of SGPP2 was significantly elevated in tumor tissue specimens, while the expression of SRD5A2 was significantly increased in normal tissue. Comparison with other relevant published studies, we comprehensive analysis and explanation of the association between the lipid metabolism-related genes with the immune microenvironment and the prognosis of PCa. We revealed the role of lipid metabolism-related genes in PCa and validated the target genes in clinical samples. Nevertheless, the specific biological functions of these genes in PCa need to be further explored.

Reprogramming of lipid metabolism is a prevalent and crucial metabolic feature that emerges during tumor evolution, allowing them to survive and further evolve in a hostile environment [37]. Through extensive exploration of aberrant lipid metabolism and tumor immunity, new breakthroughs have been made in the discovery of molecular mechanisms and metabolic adaptations, generating significant changes in antitumor therapeutic strategies [38]. Consequently, we sought to fill the gap between lipid metabolism gene status and PCa prognosis prediction. We believe these genes were involved in lipid metabolism processes, and this model may serve as a prognostic biomarker for PCa and immune microenvironment evaluation.

In conclusion, we constructed a six-gene signature associated with lipid metabolism, which was an independent prognostic factor in PCa. This six-gene signature could be recognized as a prognostic marker to reflect the lipid metabolism and immunity status of PCa.

## Data Availability

The authors declare that the data supporting the findings of the current study are provided in the article. Datasets analyzed for this work can be obtained from TCGA(https://portal.gdc.cancer.gov/), GEO (https://www.ncbi.nlm.nih.gov/geo/), MSigDB (https://www.gsea-msigdb.org/gsea/msigdb), and CIBERSORT (https://cibersort.stanford.edu/).

## Abbreviations

PCa: Prostate cancer
NMF: Nonnegative matrix factorization
TCGA: The cancer genome atlas
GEO: Gene expression omnibus
ROC: Receiver-operating characteristic
PFS: Progression-free survival
LASSO: Least absolute shrinkage and selection operator
AUC: Areas under curve
TME: Tumor microenvironment
GSVA: Gene set variation analysis
ICIs: Immune checkpoint inhibitors
IPS: Immunophenoscore
FA: Fatty acid
qRT-PCR: Quantitative real time polymerase chain reaction
TCGA-PRAD: The cancer genome atlas-prostate adenocarcinoma.

## Supplementary Materials

Supplementary Materials Figure S1. A stratified survival basis of two risk subgroups in age (a)–(b), N stage (c)–(d), clinical T stage (e)–(f), and pathologic T stage (g)–(h). Figure S2. The workflow of the present study. Table S1. Lipid metabolism-related genes. Table S2. Six pathways involved in lipid metabolism. Table S3. Clinical characteristics of prostate cancer patients from TCGA cohort. Table S4. Primer information. Table S5. 56 genes with significant prognostic differences. Table S6. 11 genes showed significant prognostic differences by cox proportional hazard analysis.
