# Supplementary material for: Discovery of Lipid Metabolism-Related Genes for Predicting Tumor Immune Microenvironment Status and Prognosis in Prostate Cancer
Source: J Oncol. 2022 Sep 5;2022:8227806. doi: 10.1155/2022/8227806 (PMC9467780; doi:10.1155/2022/8227806)
Supplement: Supplementary Materials — Figure S1. A stratified survival basis of two risk subgroups in age (a)–(b), N stage (c)–(d), clinical T stage (e)–(f), and pathologic T stage (g)–(h). Figure S2. The workflow of the present study. Table S1. Lipid metabolism-related genes. Table S2. Six pathways involved in lipid metabolism. Table S3. Clinical characteristics of prostate cancer patients from TCGA cohort. Table S4. Primer information. Table S5. 56 genes with significant prognostic differences. Table S6. 11 genes showed significant prognostic differences by cox proportional hazard analysis. [file 8227806.f1.zip › Table S2.doc]

**Table S2**: Six pathways involved in lipid metabolism.

**Pathway	Database**	**Gene Count**

Peroxisome proliferator activated receptor alpha	Reactome	119

Metabolism of lipids	Reactome	738

Transcriptional regulation of white adipocyte differentiation	Reactome	84

Sphingolipid metabolism	Reactome	89

Glycerophospholipid metabolism	KEGG	77

Fatty acid metabolism	Reactome	177

Total: 1284

Unique: 776
