# Supplementary material for: Discovery of Lipid Metabolism-Related Genes for Predicting Tumor Immune Microenvironment Status and Prognosis in Prostate Cancer
Source: J Oncol. 2022 Sep 5;2022:8227806. doi: 10.1155/2022/8227806 (PMC9467780; doi:10.1155/2022/8227806)
Supplement: Supplementary Materials — Figure S1. A stratified survival basis of two risk subgroups in age (a)–(b), N stage (c)–(d), clinical T stage (e)–(f), and pathologic T stage (g)–(h). Figure S2. The workflow of the present study. Table S1. Lipid metabolism-related genes. Table S2. Six pathways involved in lipid metabolism. Table S3. Clinical characteristics of prostate cancer patients from TCGA cohort. Table S4. Primer information. Table S5. 56 genes with significant prognostic differences. Table S6. 11 genes showed significant prognostic differences by cox proportional hazard analysis. [file 8227806.f1.zip › Table S5 (1).doc]

**Table S5: 56 genes with significant prognostic differences.**

ME1

PPARGC1A

G0S2

CYP24A1

ACSL4

PTGS2

SLCO1A2

AKR1B1

LPCAT2

PTGS1

CERK

CPNE6

SEC23A

CAV1

PIP5K1B

PTGDS

ABCC3

ACSS3

BCHE

PLA2G4A

PLA2G2D

HSD11B1

PLBD1

PTGIS

CYP2J2

FADS2

CYP27A1

CYP4B1

ETNK2

PTGES

GDPD1

PLA2G4D

ALOX15

SGPP2

ALB

STARD4

ENPP6

FABP5

ACSF2

ACER1

ACOX2

MFSD2A

GSTM4

PLEKHA2

FASN

HSD17B13

CYP4F22

GPX2

CPNE7

PLA2G2A

CPT1B

CERS1

AMACR

SRD5A2

KLF5

SLC2A4
